# Supplementary material for: Different HSP90 Inhibitors Exert Divergent Effect on Myxoid Liposarcoma In Vitro and In Vivo
Source: Biomedicines. 2022 Mar 7;10(3):624. doi: 10.3390/biomedicines10030624 (PMC8945459; doi:10.3390/biomedicines10030624)
Supplement: Supplementary file 1 [file biomedicines-10-00624-s001.zip › biomedicines-1597854-supplementary.pdf]

Supplementary figures and tables

<u>Figure S1</u>

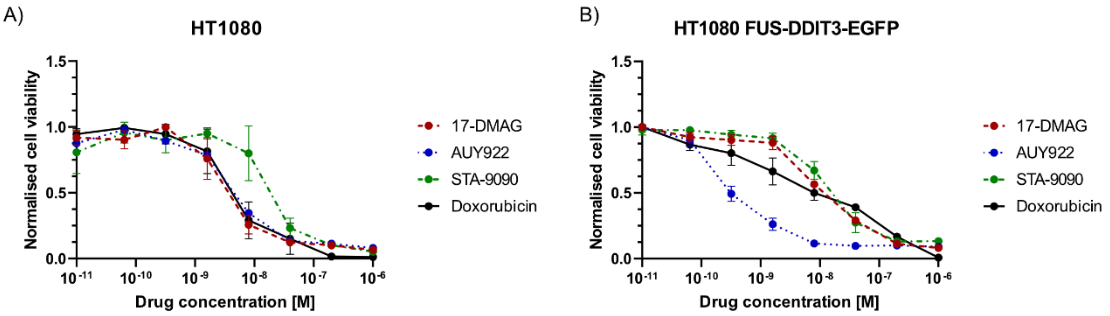

C)

Cell line	17-DMAG	AUY922	STA-9090	Doxorubicin
HT1080	3.9 nM	4.9 nM	19 nM	10 nM
HT1080 FUS-DDIT3-EGFP	13 nM	0.4 nM	17 nM	3.8 nM

Figure S1. Effect of HSP90 inhibitors *in vitro.*

A-B. Cell viability assays of the HSP90 inhibitors 17-DMAG, AUY922 and STA-9090 and the chemotherapeutic agent Doxorubicin on fibrosarcoma cell line HT1080 and HT1080-FUS-DDIT3-EGFP. Data is normalized to untreated controls. Mean +/- SD is shown.

C. Table of IC50 values of tested compounds on HT1080 and HT1080-FUS-DDIT3-EGFP.

Figure S2

A)

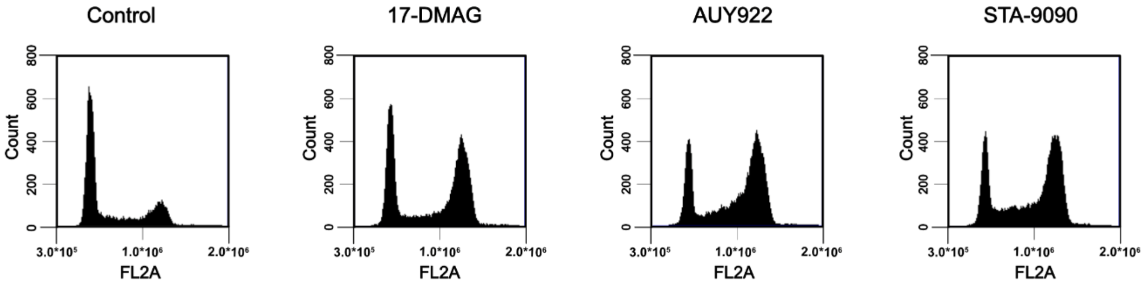

B)

Cell cycle phase	Control	17-DMAG	AUY922	STA-9090
G1	59 %	32 %	21 %	20 %
S	19 %	18 %	25 %	27 %
G2	22 %	50 %	54 %	53 %

C)

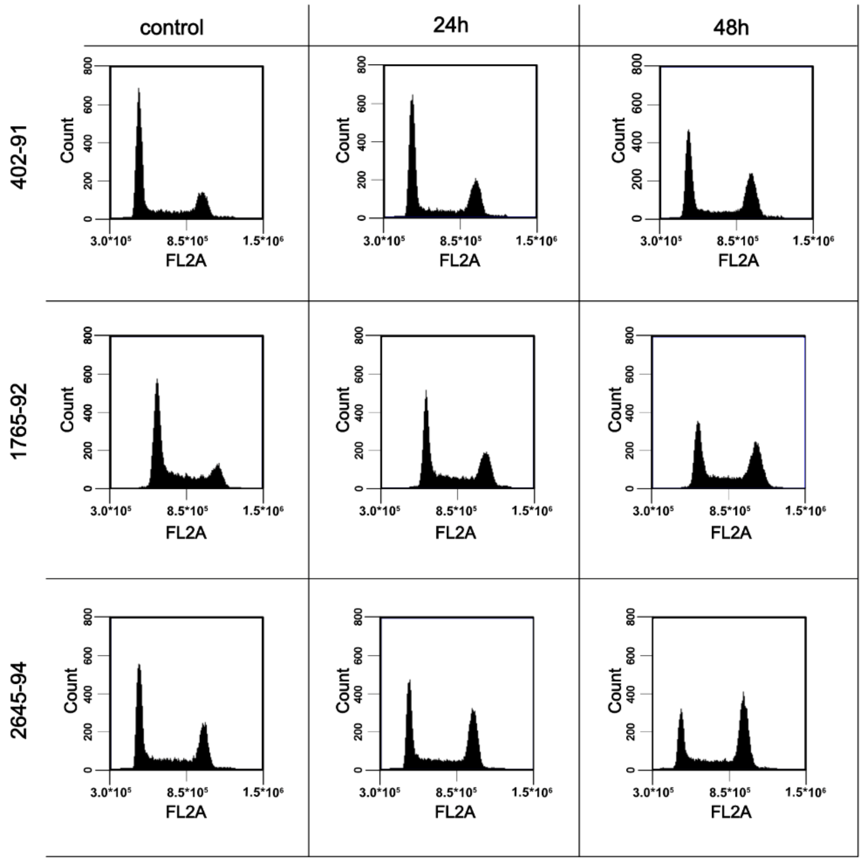

D)

Cell cycle phase	402-91			1765-92			2645-94		
	Control	24 h	48 h	Control	24 h	48 h	Control	24 h	48 h
G1	58 %	54 %	44 %	49 %	41 %	34 %	45 %	37 %	28 %
S	19 %	17 %	20 %	31 %	27 %	26 %	24 %	23 %	23 %
G2	23 %	29 %	36 %	20 %	32 %	40 %	31 %	40 %	49 %

Figure S2. Cell cycle analysis using flow cytometry.

A. Raw data for Fig **1F**. Plots showing PI-intensity (FL2A) vs count. First peak indicates cells in G1 phase, second peak cells in G2 phase and the intermediate region cells in S phase.

B. Raw data for Fig **1F**. Table summarizing cell cycle distribution upon different treatment conditions.

C. Raw data for Fig **1G**. Plots showing PI-intensity (FL2A) vs count. First peak indicates cells in G1 phase, second peak cells in G2 phase and the intermediate region cells in S phase.

D. Raw data for Fig **1G.** Table summarizing cell cycle distribution upon different treatment conditions.

Figure S3

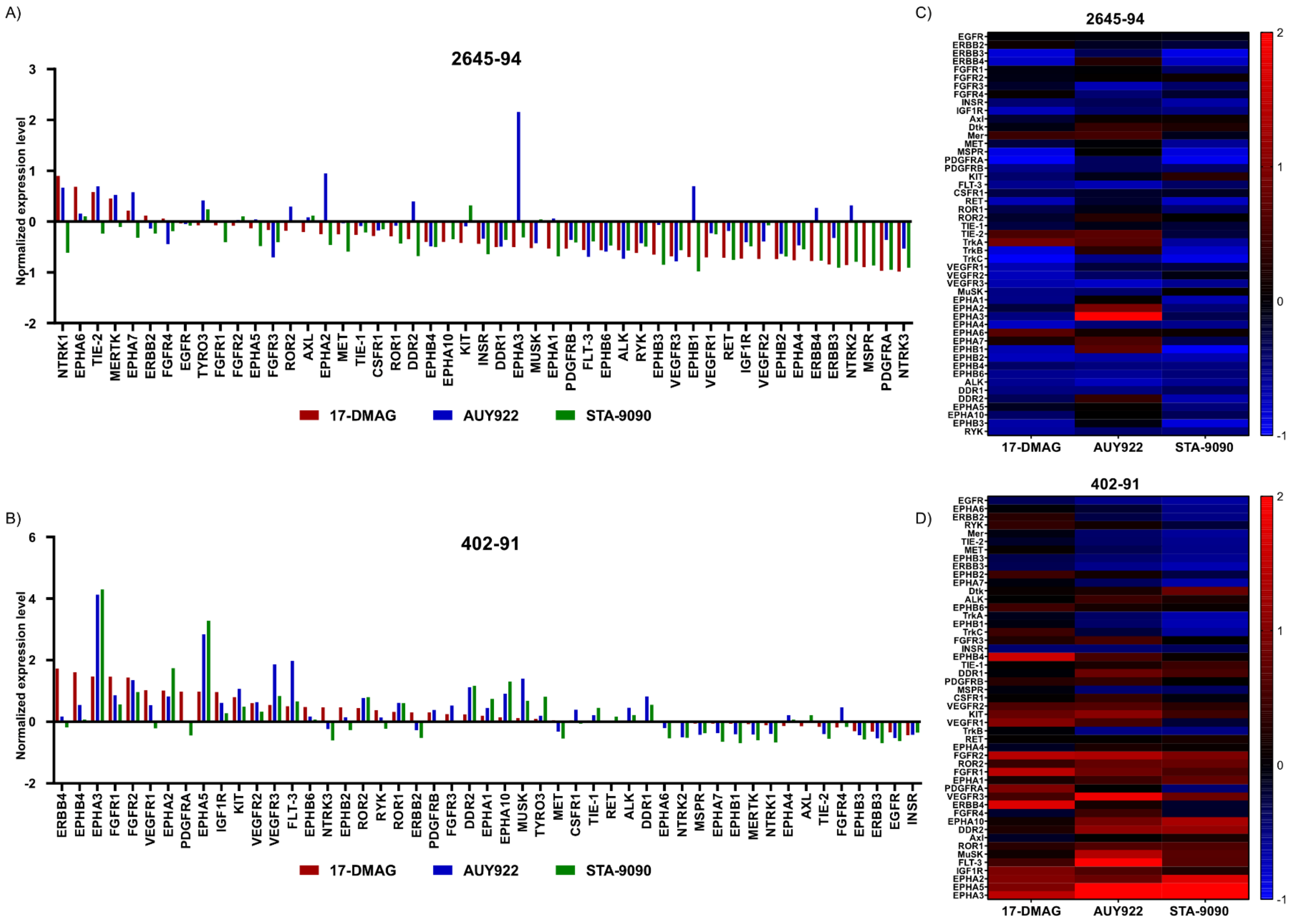
A)
2645-94
Normalized expression level
3
2
1
0
-1
-2
NTRK1 EPHA6 TIE-2 MERTK EPHA7 ERBB2 FGFR4 EGFR TYRO3 FGFR1 FGFR2 EPHA5 FGFR3 ROR2 AXL EPHA2 MET TIE-1 CSFR1 ROR1 DDR2 EPHB4 EPHA10 KIT INSR DDR1 EPHA3 MUSK EPHA1 PDGFRB FLT-3 EPHB6 ALK RYK EPHB3 VEGFR3 EPHB1 VEGFR1 RET IGF1R VEGFR2 EPHB2 EPHA4 ERBB4 ERBB3 NTRK2 MSPR PDGFRA NTRK3
17-DMAG
AUY922
STA-9090
B)
402-91
Normalized expression level
6
4
2
0
-2
ERBB4 EPHB4 EPHA3 FGFR1 FGFR2 VEGFR1 EPHA2 PDGFRA EPHA5 IGF1R KIT VEGFR2 VEGFR3 FLT-3 EPHB6 NTRK3 EPHB2 ROR2 RYK ROR1 ERBB2 PDGFRB FGFR3 DDR2 EPHA1 EPHA10 MUSK TYRO3 MET CSFR1 TIE-1 RET ALK DDR1 EPHA6 NTRK2 MSPR EPHA7 EPHB1 MERTK NTRK1 EPHA4 AXL TIE-2 FGFR4 EPHB3 ERBB3 EGFR INSR
17-DMAG
AUY922
STA-9090
C)
2645-94
2
1
0
-1
17-DMAG
AUY922
STA-9090
D)
402-91
2
1
0
-1
17-DMAG
AUY922
STA-9090

Figure S3. Phospho-RTK analysis of 49 phospho-RTKs in MLS2645-94 and 402-91 upon HSP90 inhibition with 100 nM 17-DMAG, 50 nM AUY922 or 80 nM STA-9090 for 24 h. Data presented as bar charts of mean relative expression of two measurements, compared with untreated control (**A-B**) and heat maps, where blue color indicates downregulation and red color indicates upregulation (**C-D**).

Figure S4

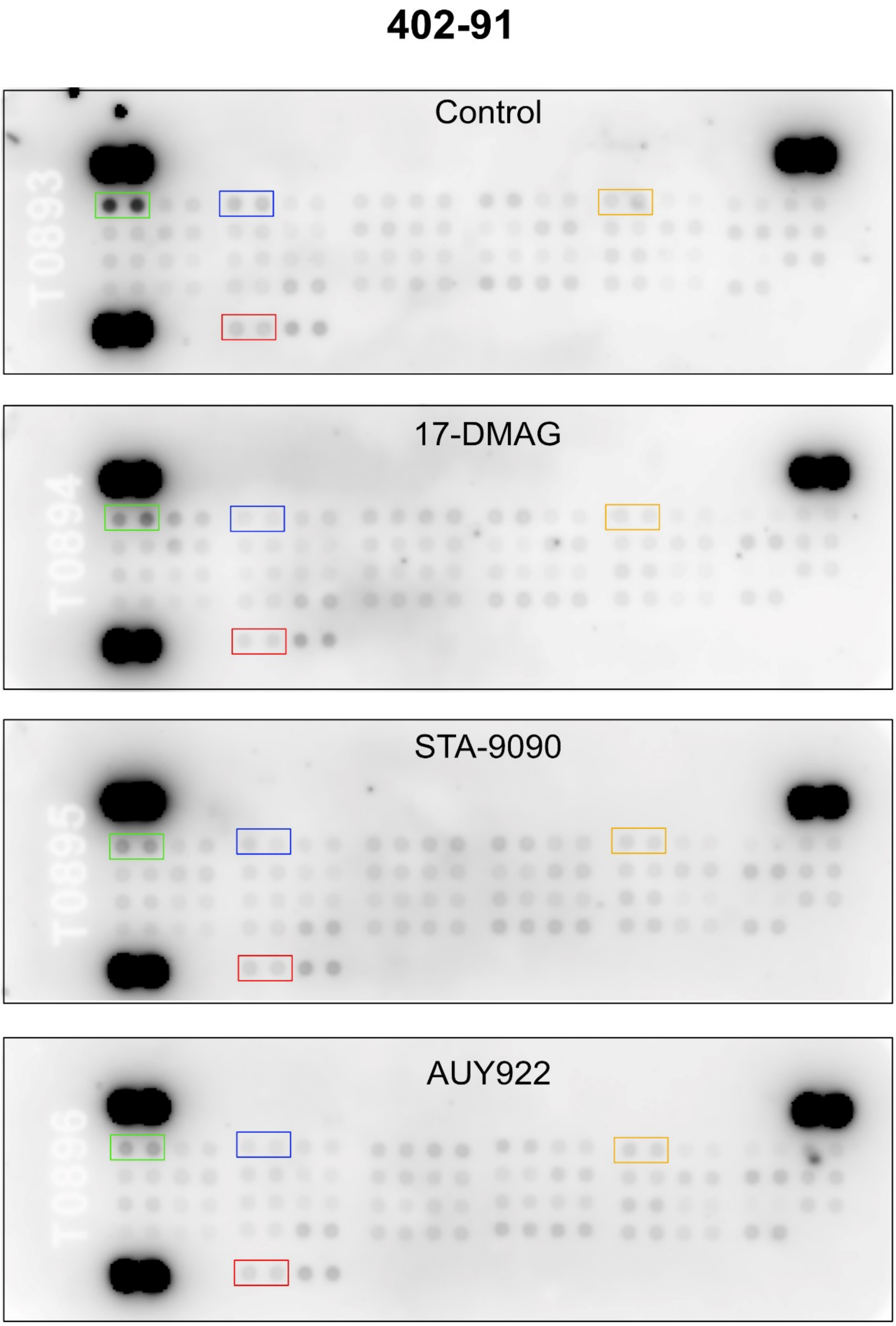

Figure S4. Raw data for pRTK analysis of MLS cell line 402-91 upon HSP90 inhibition with 100 nM 17-DMAG, 50 nM AUY922 or 80 nM STA-9090 for 24 h**.** Green box = EGFR. Blue box = ERBB3. Orange box = INSR. Red box = EPHB3.

Figure S5

2645-94

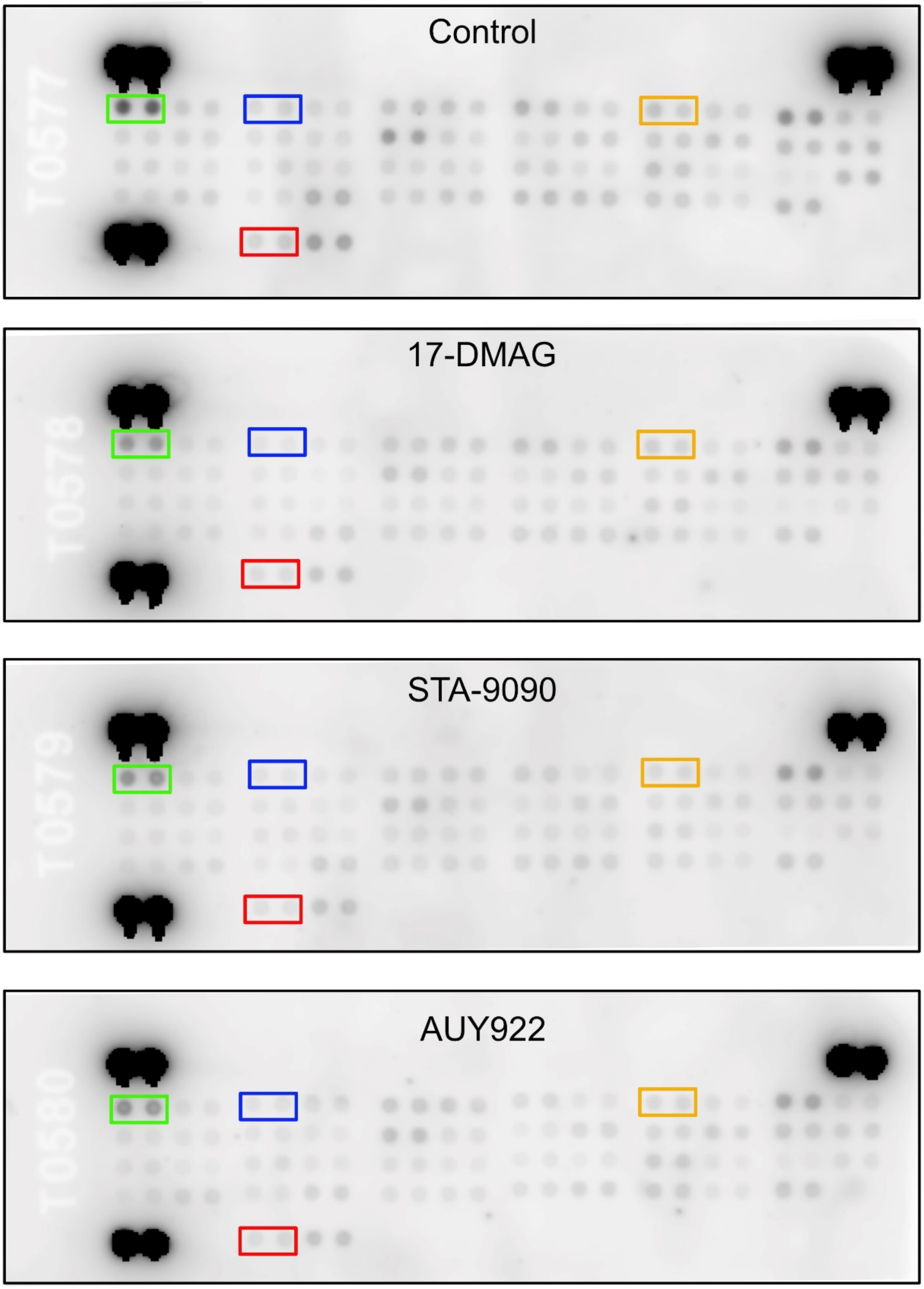

Figure S5. Raw data for pRTK analysis of MLS cell line 2645-94 upon HSP90 inhibition with 100 nM 17-DMAG, 50 nM AUY922 or 80 nM STA-9090 for 24 h**.** Green box = EGFR. Blue box = ERBB3. Orange box = INSR. Red box = EPHB3.

Figure S6

A)

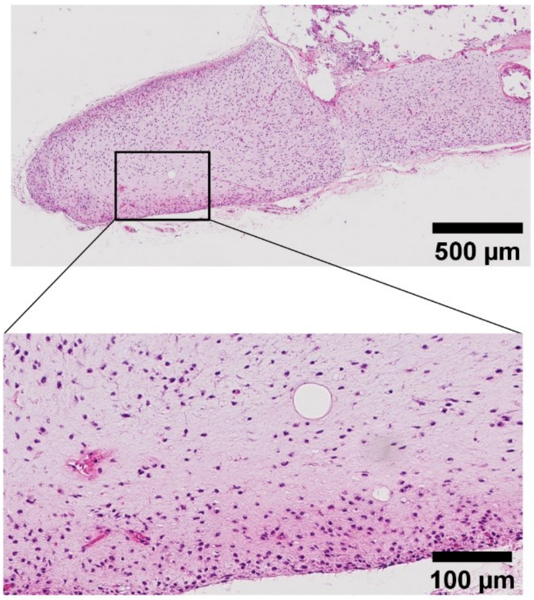

B)

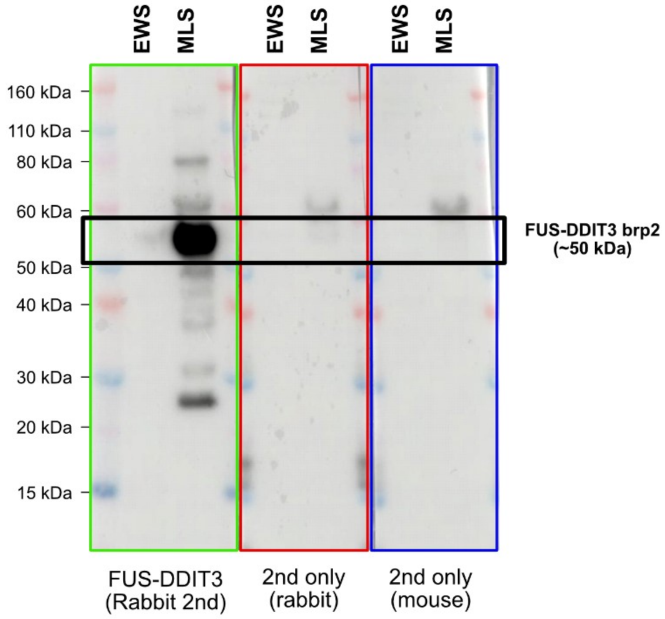

C)

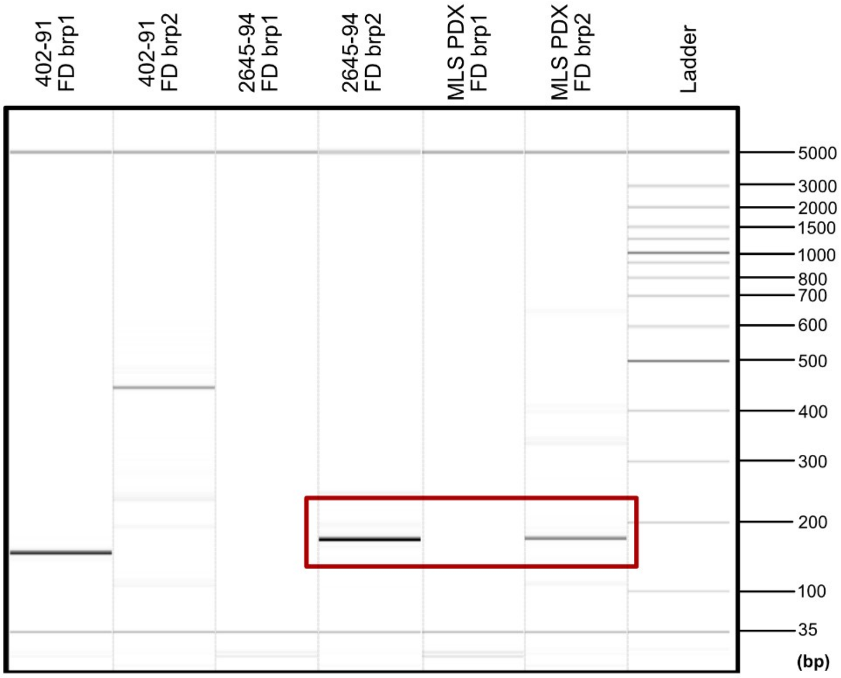

Figure S6. MLS PDX model.

A. H&E staining of an MLS PDX tumor section.

B. Western blot, using a DDIT3 rabbit primary antibody, detecting FUS-DDIT3. In the MLS PDX, a signal is detected at approximately 50 kDa, indicating a type 2 FUS-DDIT3 fusion oncogene. An EWS PDX, used as negative control, does not give any signal. Incubation with secondary antibodies only (Rabbit + Mouse) give an unspecific signal at approximately 60 kDa.

C. Analysis of PCR products of the MLS PDX using primers against type 1 and type 2 FUS-DDIT3 fusion oncogenes using Fragment Analyzer. MLS cell lines 402-91 and 2645-94, harboring a type 1 and type 2 fusion respectively, were used as positive controls. The MLS PDX harbors a type 2 FUS-DDIT3 oncogene (167 bp).

<u>Figure S7</u>

A)

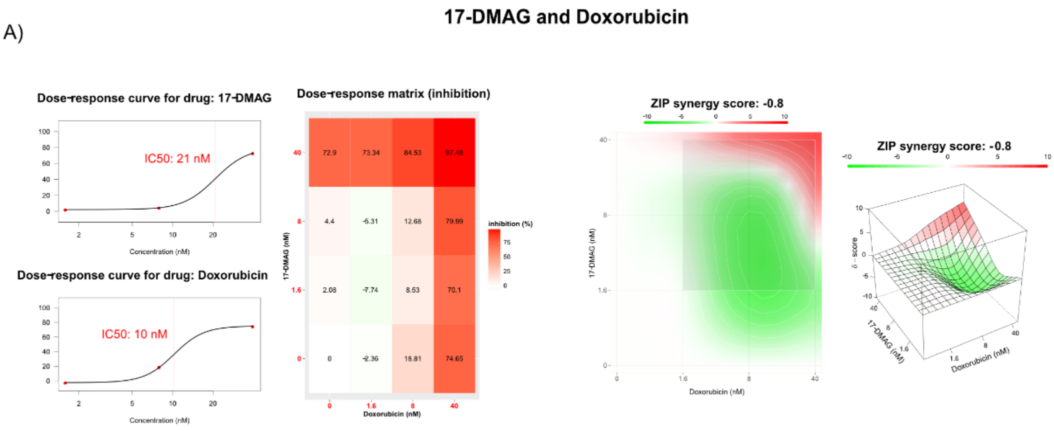

B)

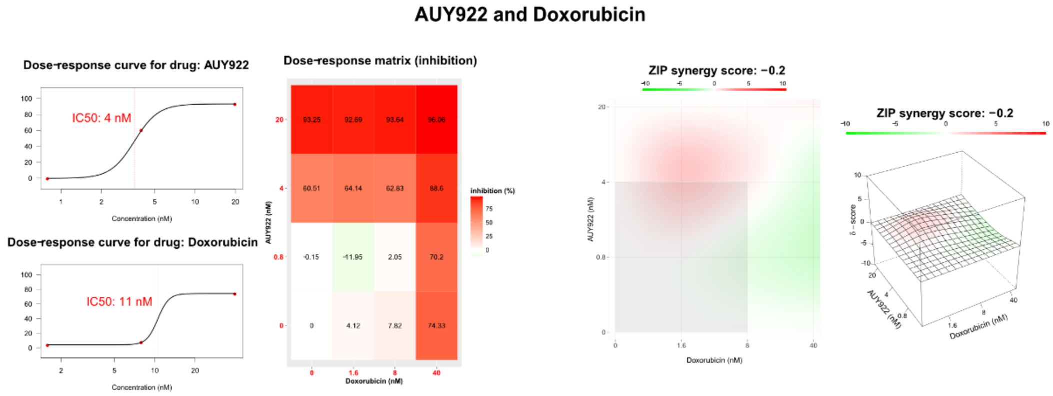

C)

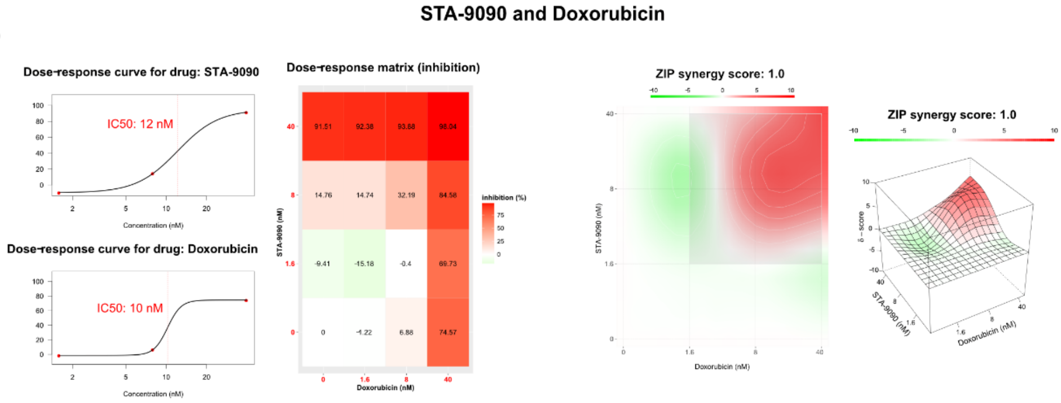

Figure S7. Combination cell viability assays, doxorubicin and HSP90 inhibition

A-C. Dose-response curves, dose-response matrixes and 2D/3D synergy matrixes on MLS 402-91 treated with 17-DMAG (**A**), AUY922 (**B**) or STA-9090 (**C**) in combination with doxorubicin at indicated doses. IC50 values are plotted in red text for each drug in the dose-response curves. Mean ZIP synergy score is displayed above 2D/3D synergy matrixes. Negative values of ZIP score indicate antagonism, 0 indicates additive drug effect and positive values indicate synergistic effect. The dose response curves and 3D synergy matrixes are also displayed in Fig **5A**, **5C** and **5E**.

Figure S8

A)

17-DMAG and Trabectidin

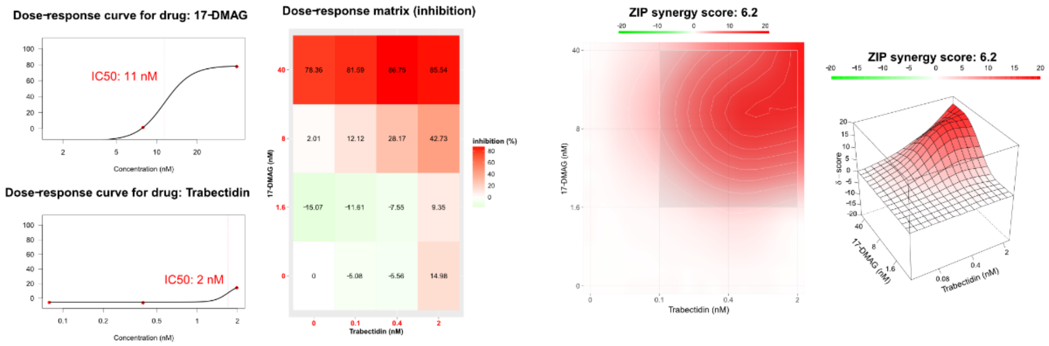

B)

AUY922 and Trabectidin

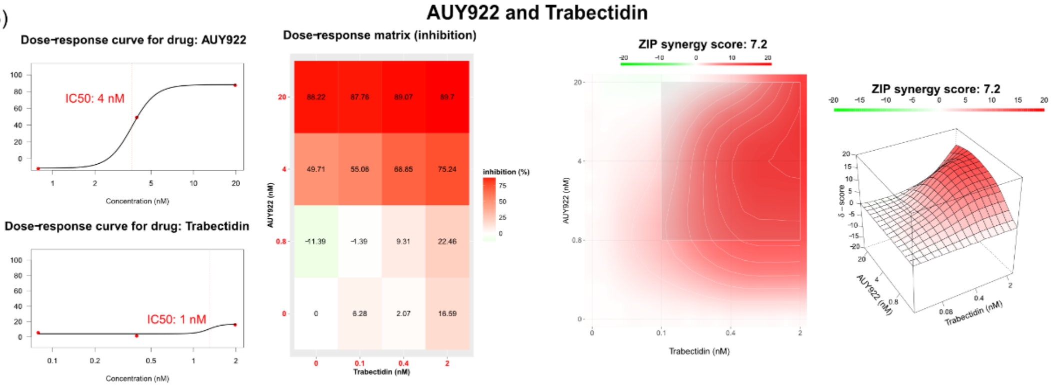

C)

STA-9090 and Trabectidin

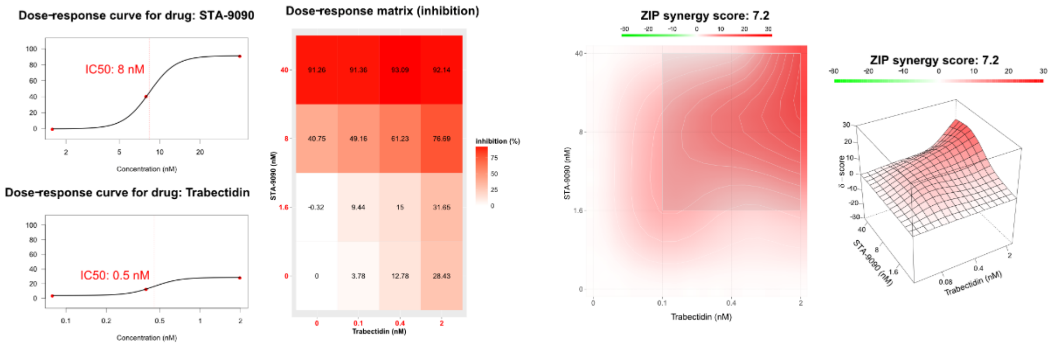

Figure S8. Combination cell viability assays, lower dose of trabectidin and HSP90 inhibition

A-C. Dose-response curves, dose-response matrixes and 2D/3D synergy matrixes on MLS 402-91 treated with 17-DMAG (**A**), AUY922 (**B**) or STA-9090 (**C**) in combination with trabectidin at indicated doses. IC50 values are plotted in red text for each drug in the dose-response curves. Mean ZIP synergy score is displayed above 2D/3D synergy matrixes. Negative values of ZIP score indicate antagonism, 0 indicates additive drug effect and positive values indicate synergistic effect. The dose response curves and 3D synergy matrixes are also displayed in Fig **5B**, **5D** and **5F**.

Figure S9

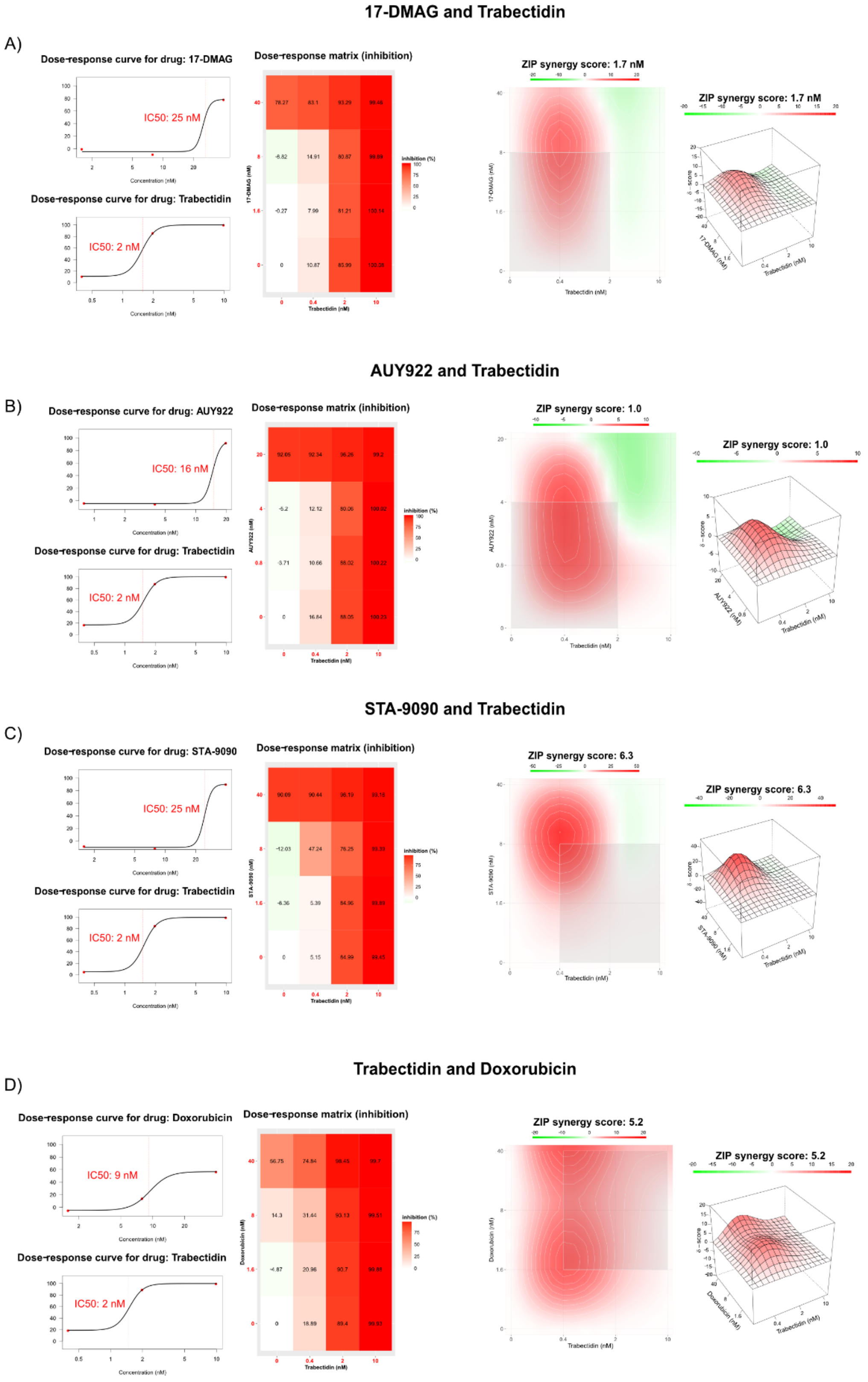

17-DMAG and Trabectidin
A)
Dose-response curve for drug: 17-DMAG
IC50: 25 nM
Dose-response curve for drug: Trabectidin
IC50: 2 nM
Dose-response matrix (inhibition)
ZIP synergy score: 1.7 nM
ZIP synergy score: 1.7 nM
AUY922 and Trabectidin
B)
Dose-response curve for drug: AUY922
IC50: 16 nM
Dose-response curve for drug: Trabectidin
IC50: 2 nM
Dose-response matrix (inhibition)
ZIP synergy score: 1.0
ZIP synergy score: 1.0
STA-9090 and Trabectidin
C)
Dose-response curve for drug: STA-9090
IC50: 25 nM
Dose-response curve for drug: Trabectidin
IC50: 2 nM
Dose-response matrix (inhibition)
ZIP synergy score: 6.3
ZIP synergy score: 6.3
Trabectidin and Doxorubicin
D)
Dose-response curve for drug: Doxorubicin
IC50: 9 nM
Dose-response curve for drug: Trabectidin
IC50: 2 nM
Dose-response matrix (inhibition)
ZIP synergy score: 5.2
ZIP synergy score: 5.2

Figure S9. Combination cell viability assays, higher dose of trabectidin and HSP90 inhibition

A-C. Dose-response curves, dose-response matrixes and 2D/3D synergy matrixes on MLS 402-91 treated with 17-DMAG (**A**), AUY922 (**B**) or STA-9090 (**C**) in combination with trabectidin at indicated doses. IC50 values are plotted in red text for each drug in the dose-response curves. Mean ZIP synergy score is displayed above 2D/3D synergy matrixes. Negative values of ZIP score indicate antagonism, 0 indicates additive drug effect and positive values indicate synergistic effect.

Table S1. Morphological evaluation of H&E stained PDX tumors

Treatment	Morphological analysis
Control	MLS morphology. Peripheral crowding. Poorly developed capillary network. Some debris. No lipoblasts. No round cell component
Control	MLS morphology. Peripheral crowding. Poorly developed capillary network. Few lipoblasts. No round cell component
Control	MLS morphology. Peripheral crowding. Sparse capillary network. Some debris. Few lipoblasts. No round cell component
Control	MLS morphology. Poorly developed capillary network. Few lipoblasts. Some debris. No round cell component
Control	MLS morphology. Poorly developed capillary network. Some debris. No lipoblasts. No round cell component
17-DMAG	MLS morphology. Small and hypocellular. No peripheral crowding. Some lipoblasts. No round cell component
17-DMAG	MLS morphology. Small and hypocellular. No peripheral crowding. Poorly developed capillary network. Some debris. No lipoblasts. No round cell component
17-DMAG	MLS morphology. Hypocellular. Some peripheral crowding. Poorly developed capillary network. Abundant debris and necrosis. No lipoblasts. No round cell component
17-DMAG	MLS morphology. Peripheral crowding. Possibly more elaborate capillary network (difficult to assess). Abundant lipoblasts. Debris and necrosis. No round cell component
AUY922	MLS morphology. Cellular. No peripheral crowding. Elaborate capillary and vascular network. Diffuse bleeding around capillaries and hemorrhage. No lipoblasts. No round cell component
AUY922	MLS morphology. Small. No peripheral crowding. Poorly developed capillary network. No lipoblasts. No round cell component
AUY922	MLS morphology. A bit hypocellular. Some peripheral crowding. Poorly developed capillary network. Few lipoblasts. No round cell component

AUY922	MLS morphology. Cellular. Peripheral crowding. More elaborate capillary network. Abundant lipoblasts. No round cell component, but some cells larger.
AUY922	MLS morphology. Cellular. No peripheral crowding. Elaborate capillary network. Some debris. Few lipoblasts. No round cell component
AUY922	MLS morphology. Peripheral crowding. Poorly developed capillary network. More lipoblasts. No round cell component
AUY922	MLS morphology. Cellular. Peripheral crowding. Sparse capillary network. Few lipoblasts. No round cell component
STA-9090	MLS morphology. Peripheral crowding. Possibly more elaborate capillary network (difficult to assess). Abundant lipoblasts. No round cell component
STA-9090	MLS morphology. No peripheral crowding. Poorly developed capillary network. Some debris. More lipoblasts. No round cell component
STA-9090	MLS morphology. Peripheral crowding. More prominent capillary network. Few lipoblasts. No round cell component
STA-9090	MLS morphology. Some peripheral crowding. Poorly developed capillary network. Some debris and necrosis. More lipoblasts. No round cell component
AUY922 → 17-DMAG	MLS morphology. Hypocellular. No peripheral crowding. Poorly developed capillary network. Some debris. No lipoblasts. No round cell component
AUY922 → 17-DMAG	MLS morphology. No peripheral crowding. Poorly developed capillary network. Some debris. No lipoblasts. No round cell component
AUY922 → 17-DMAG	MLS morphology. Some peripheral crowding. Poorly developed capillary network. More debris and necrosis. Few lipoblasts. No round cell component
AUY922 → 17-DMAG	MLS morphology. Peripheral crowding. Poorly developed capillary network. More debris. More lipoblasts. No round cell component
STA-9090 2 weeks	MLS morphology. Peripheral crowding. More elaborate capillary network and some dilated vessels. Abundant lipoblasts. No round cell component
STA-9090 2 weeks	MLS morphology. Peripheral crowding. More elaborate capillary network and some dilated vessels. Abundant lipoblasts. No round cell component

Table S2. Antibodies used for Western blot

Target	Company/product nr	Species	Dilution/Block	Size
DDIT3	Proteintech 15204-1-Ap	Rabbit	1:1000 milk	FUSDDIT3: 50-100 kDa<br>DDIT3: 30 kDa
EGFR	Santa Cruz sc-03	Rabbit	1:500 BSA	150-175 kDa
p-EGFR	Cell signaling #2236	Mouse	1:1000 BSA	150-175 kDa
ERBB3	Santa Cruz sc-285	Rabbit	1:100 BSA	150-170 kDa
p-ERBB3	Cell signalling #4791	Rabbit	1:1000 BSA	150-170 kDa
MEK1/2	Cell signalling #9122	Rabbit	1:1000 BSA	45 kDa
p-MEK1/2	Cell signalling #4695	Rabbit	1:1000 BSA	45 kDa
ERK1/2	Cell signalling #4695	Rabbit	1:1000 BSA	44 kDa
p-ERK1/2	Cell signalling #4376	Rabbit	1:1000 BSA	44 kDa
AKT	Cell signalling #2967	Mouse	1:1000 BSA	60 kDa
p-AKT	Cell signalling #4051	Mouse	1:1000 BSA	60 kDa
HSP90	ABIN361665	Mouse	1:2000 BSA	90 kDa
HSP70	Santa Cruz sc-24	Mouse	1:1000 BSA	70 kDa
Caspase 3	Santa Cruz sc-7272	Mouse	1:1000 BSA	34 kDa
Cleaved caspase 3	Cell signalling #9664	Rabbit	1:1000 BSA	17/19 kDa
Vinculin	Sigma V4505	Mouse	1:1000 BSA	117 kDa
GAPDH	Proteintech 60004-1-Ig	Mouse	1:50 000 BSA	36 kDa

Table S3. Primers used and PCR product length

Gene	Forward primer sequence	Reverse primer sequence	Length
FUS-DDIT3 bp1	GACCGTGGTGGCTTCAATAA	CAGTGTCCCGAAGGAGAAA	147 bp
FUS-DDIT3 bp2	AGCAGAACCAGTACAACAGC	CCCGAAGGAGAAAGGCAATG	167 bp

Table S4. Summary of *In vitro* responses to HSP90 inhibition

In vitro response								
**Compound**	**MLS cell lines**	**IC50 (72 h)**	**Cell cycle inhibition**	**Induction of Cleaved caspase 3**	**Growth receptor response**	**MAPK signaling**	**Synergy score (doxorubicin)**	**Synergy score (trabectidin)**
17-DMAG	402-91 (type 1 fusion)	17 nM	G2/S inhibition	+	Downregulation of ERBB3 (phosphorylation) and EGFR (total protein)	Downregulation of MAPK and AKT	-0.8	6.2
	1765-92 (type 6 fusion)	3 nM	G2/S inhibition	++	Downregulation of ERBB3 (phosphorylation and total protein) and EGFR (total protein)	Downregulation of MAPK and AKT	N/A	N/A
	2645-94 (type 2 fusion)	4.2 nM	G2/S inhibition	+	Downregulation of ERBB3 (phosphorylation) and EGFR (total protein)	Downregulation of MAPK and AKT	N/A	N/A
AUY922	402-91 (type 1 fusion)	5.3 nM	G2/S inhibition	+++	Downregulation of ERBB3 (phosphorylation) and EGFR (total protein)	Upregulation of ERK, no regulation of AKT	-0.2	7.2
	1765-92 (type 6 fusion)	0.1 nM	N/A	++	Downregulation of ERBB3 (phosphorylation and total protein) No regulation of EGFR	Upregulation of ERK, no regulation of AKT	N/A	N/A
	2645-94 (type 2 fusion)	15 nM	N/A	++	Downregulation of ERBB3 (phosphorylation) and EGFR (total protein)	Downregulation of MAPK and AKT	N/A	N/A
STA-9090	402-91 (type 1 fusion)	24 nM	G2/S inhibition	+++	Downregulation of ERBB3 (phosphorylation and total protein) and EGFR (total protein)	Downregulation of MAPK and AKT	1.0	7.2
	1765-92 (type 6 fusion)	4.9 nM	N/A	++	Downregulation of ERBB3 (phosphorylation and total protein), No regulation of EGFR	Downregulation of MAPK and AKT	N/A	N/A
	2645-94 (type 2 fusion)	15 nM	N/A	++	Downregulation of ERBB3 (phosphorylation) and EGFR (total protein)	Downregulation of MAPK and AKT	N/A	N/A

+ / ++ / +++ indicates <5 / 5-10 / >10 times elevation of protein expression levels compared to control. N/A indicates that experiment was not performed.

Table S5. Summary of *In vivo* responses to HSP90 inhibition

In vivo response				
**Compound**	**Tumor growth**	**Toxicity**	**Morphology**	**Other**
17-DMAG	Growth inhibition	No toxicity	Hypocellular. More lipoblasts	Shows treatment effect on tumors not responding to AUY922
AUY922	No growth inhibition	No toxicity	Hypercellular, pleomorphic morphology	-
STA-9090	No growth inhibition	Slight weight loss	No difference to controls	Short term treatment with toxic doses leads to tumor cell death and induction of lipoblasts
